# What is complex allometry?

**DOI:** 10.1242/bio.060148

**Published:** 2023-12-21

**Authors:** Gary C. Packard

**Affiliations:** Department of Biology, Colorado State University, Fort Collins, CO 80523, USA

**Keywords:** Allometry, Complex allometry, Nonlinear allometry, Metabolic allometry

## Abstract

*Complex allometry* describes a smooth, curvilinear relationship between logarithmic transformations of a biological variable and a corresponding measure for body size when the observations are displayed on a bivariate graph with linear scaling. The curvature in such a display is commonly captured by fitting a quadratic equation to the distribution; and the quadratic term is typically interpreted, in turn, to mean that the mathematically equivalent equation for describing the arithmetic distribution is a two-parameter power equation with an exponent that changes with body size. A power equation with an exponent that is itself a function of body size is virtually uninterpretable, yet numerous attempts have been made in recent years to incorporate such an exponent into theoretical models for the evolution of form and function in both plants and animals. However, the curvature that is described by a quadratic equation fitted to logarithms usually means that an explicit, non-zero intercept is required in the power equation describing the untransformed distribution — not that the exponent in the power equation varies with body size. Misperceptions that commonly accompany reports of complex allometry can be avoided by using nonlinear regression to examine untransformed data.

## Teaser

**Summary:** Complex bivariate allometry in logarithmic domain is caused by a requirement for an explicit intercept in the power equation describing untransformed observations.

## INTRODUCTION

*Complex allometry* is a pattern of bivariate variation that follows a smooth, curvilinear path when logarithmic transformations of a biological variable (Y) and a corresponding measure for body size (X) are displayed on a graph with linear coordinates (or alternatively, when measurements for the untransformed variables are displayed on a graph with logarithmic scaling) (Strauss, 1993). Distributions like this have occasionally been modeled as exponential functions (e.g. Streicher et al., 2012) or growth curves (e.g. Laird et al., 1968; Jolicoeur, 1989). However, observations typically appear in graphical display to approximate a parabolic curve, so the relationship between the transformed variables is usually characterized in contemporary research as a quadratic polynomial,
(1)

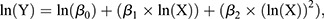
where *β_0_*, *β_1_*, and *β_2_* are fitted parameters (e.g. Kozlowski and Konarzewski, 2005; Moran and Wells, 2007; Clarke et al., 2010; Kolokotrones et al., 2010; Deeds et al., 2011; Ehnes et al., 2011; Müller et al., 2012; Glazier et al., 2013; Bueno and López-Urrutia, 2014; Lemaître et al., 2014; Douhard et al., 2016; Tidière et al., 2017). The quadratic term in the equation accounts for the curvature in log domain (Deeds et al., 2011; Glazier, 2022) and generally is interpreted to mean that the equivalent equation for describing the original, untransformed data is a two-parameter power function with an exponent that changes with body size (e.g. Strauss, 1993; Kozlowski and Konarzewski, 2005; Moran and Wells, 2007; Clarke et al., 2010; Kolokotrones et al., 2010; Müller et al., 2012; Glazier et al., 2013; Bueno and López-Urrutia, 2014; Lemaître et al., 2014; Douhard et al., 2016; Tidière et al., 2017). The concept of a changing exponent in a simple power equation has been widely embraced by workers studying allometric variation (Strauss, 1993) but the notion has arguably had its greatest impact in research on the scaling of metabolic rates (e.g. Kozlowski and Konarzewski, 2005; Moran and Wells, 2007; Clarke et al., 2010; Kolokotrones et al., 2010; Müller et al., 2012; Glazier et al., 2013; Bueno and López-Urrutia, 2014). Indeed, the belief that the exponent is a function of body size in statistical models for describing metabolic allometry has led in some instances to revisions of existing theory for the evolution of form and function and in other instances to the formulation of new theory (Savage et al., 2008; Brown and Sibly, 2012; Banavar et al., 2014; Glazier, 2014; Kozlowski et al., 2020).

Although leaders in the field of allometry research promote the use of quadratic equations to describe distributions that are curvilinear in logarithmic domain (e.g. Glazier, 2022; Mortola, 2023), the concept and methods of complex allometry are seriously flawed. For example, back-transforming a quadratic equation from the logarithmic domain (Eqn 1) to the arithmetic scale yields a ‘two-parameter’ power equation with three parameters and two predictor variables,
(2)

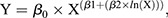
where *β_0_*, *β_1_*, and *β_2_* are the parameters from the equation fitted to logarithms (MacKay, 2011; Müller et al., 2012; Bueno and López-Urrutia, 2014; Packard, 2015a, 2017a,b). The two parameters in the exponent for the equation, together with a lack of independence for the two predictors, render the model uninterpretable (Packard, 2015a). Moreover, by focusing an analysis on transformations, investigators almost invariably fail to appreciate the actual relationship between the untransformed variables or the cause for curvilinearity in log domain (Bales, 1996; Sartori and Ball, 2009; Packard, 2017b). This third issue is critical, because an understanding of the biological significance of allometric variation cannot be achieved without first acquiring an accurate appreciation for pattern in the data (White and Seymour, 2005).

How can these issues be resolved? Here I use data from an investigation of metabolic allometry in Madagascan cockroaches (*Gromphadorhina portentosa*) to illustrate the underlying problem of complex allometry and to describe how the problem can be circumvented altogether.

## RESULTS

**Fig. 1.**
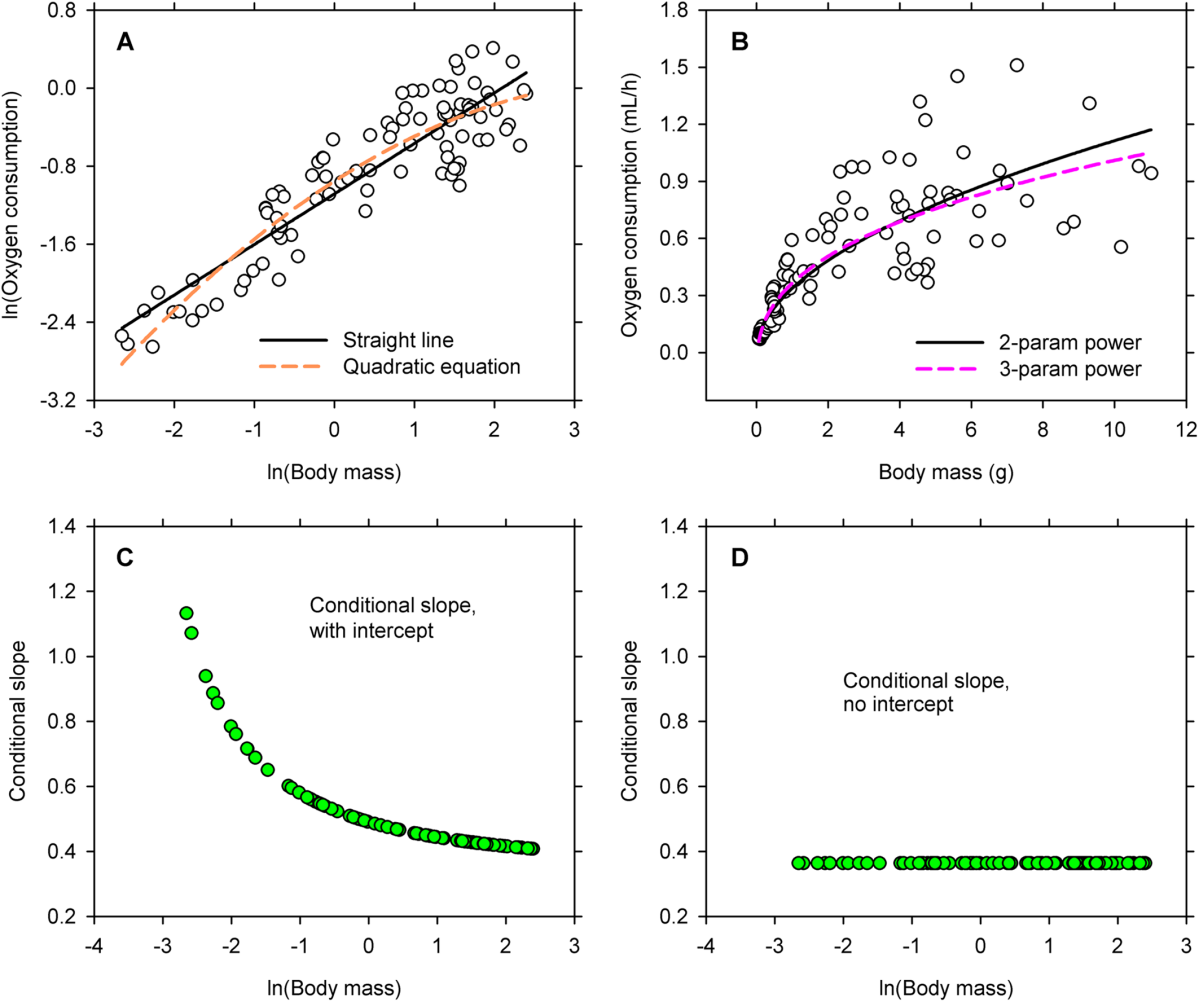
**Allometric variation in oxygen consumption of Madagascan cockroaches at 28°C.** (A) A straight line and quadratic polynomial were fitted to logarithmic transformations of oxygen consumption and body mass. (B) Two- and three-parameter power equations were fitted to untransformed observations for oxygen consumption and body mass. (C) Conditional slopes for the three-parameter power equation in logarithmic form. (D) Conditional slopes for the transformed power equation when the intercept is set at zero. Other values in the equation are unchanged, so values for the conditional slope differ from the slope for the straight line fitted to transformations (Table 1).

I used WebPlotDigitizer (https://automeris.io/WebPlotDigitizer) to capture 92 pairs of observations for metabolic rate (measured as oxygen consumption) and body mass of Madagascan cockroaches from Fig. 1A of the article by Streicher et al. (2012). The data set comprises an ontogenetic series ranging from small nymphs to large adults (Table S1). The measurements then were transformed to natural logarithms and displayed on a bivariate graph (Fig. 1A). A straight line and a quadratic polynomial were fitted to the distribution by ordinary least squares (Smith, 2009; Kilmer and Rodríguez, 2017), and the resulting models were compared by Akaike's Information Criterion, or AIC (Burnham and Anderson, 2002). As a rule of thumb, an AIC that differs from AIC for the best model (i.e. the lowest) by no more than 2 identifies a model that is equivalent to the best model, and an AIC that differs from the reference by 3–6 identifies a plausible alternative to the best model (Richards, 2005; Burnham et al., 2011; Richards et al., 2011; Murtaugh, 2014). Models with AIC >7 have little to recommend them. The straight line fitted here is, of course, the transformed equation of simple allometry promoted by Huxley (1932).

**Table 1.**
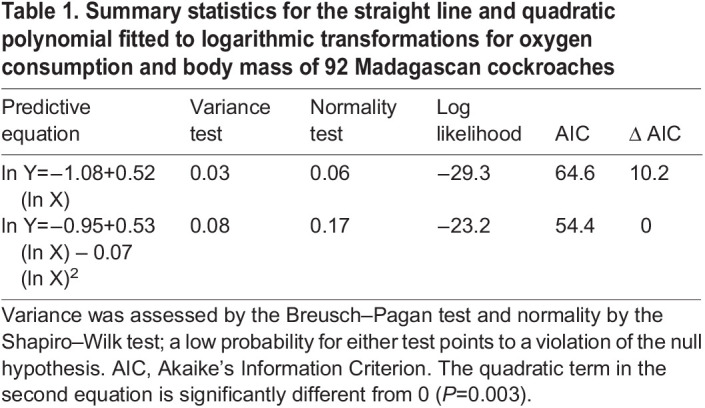
Summary statistics for the straight line and quadratic polynomial fitted to logarithmic transformations for oxygen consumption and body mass of 92 Madagascan cockroaches

AIC for the quadratic model is lower than that for the straight line by 10.04 (Table 1), thereby indicating that the quadratic is the better fit (Richards, 2005; Burnham et al., 2011; Richards et al., 2011). Moreover, all the coefficients in the quadratic model are significant sources of variation by *t*-test (*P*≤0.003), and the model satisfies assumptions for homoscedasticity and normality (Table 1). These findings would be reported by many investigators as an example of complex bivariate allometry, and the quadratic term would be taken to mean that the exponent is a varying function of body size in a two-parameter power equation describing the untransformed distribution (Strauss, 1993).

It is always better to study untransformed data when it is possible to do so (Finney, 1989; Menge et al., 2018). I accordingly displayed the untransformed data on a bivariate graph (Fig. 1B) and submitted them to analysis using the Model Procedure in SAS 9.4 (SAS Institute Inc., 2004). I fitted regression models for straight lines and power functions, with all the models having lognormal, heteroscedastic error (https://support.sas.com/documentation/cdl/en/etsug/60372/HTML/default/viewer.htm#etsug_model_sect045.htm). Thus, the error structure in the models aligned with that of a model formed by back-transforming an equation fitted to logarithms (Packard, 2020; 2023). This an important point because many investigators are under the erroneous impression that nonlinear regression can only fit models with normal, homoscedastic error.

Power equations are better than straight lines for modeling the untransformed data, and the three-parameter model is marginally better than the two-parameter model (Table 2). Nevertheless, the intercept for the three-parameter equation is not significantly different from zero by *t*-test (*P*=0.14), and both the two-parameter and the three-parameter functions provide reasonable descriptions for the observations in graphical display (Fig. 1B). What is noteworthy, however, is the allometric exponent in the power models: it is a constant in both equations (Table 2).

**Table 2.**
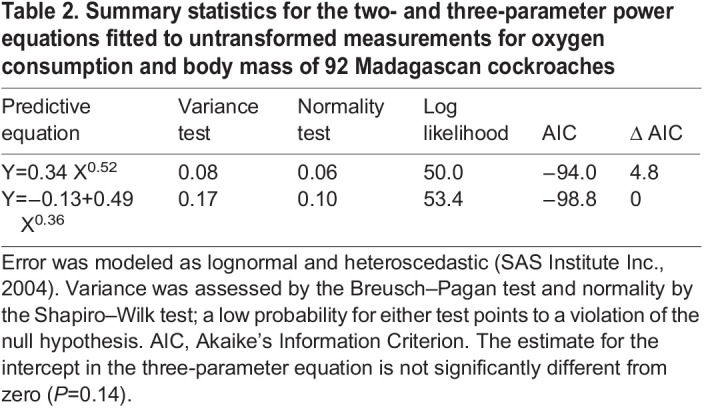
Summary statistics for the two- and three-parameter power equations fitted to untransformed measurements for oxygen consumption and body mass of 92 Madagascan cockroaches

A case can be made for accepting the two-parameter model on grounds that the intercept does not enter the three-parameter model at a significant level. On the other hand, a case can also be made for accepting the three-parameter model on grounds that it captures slightly more of the information in the data than does the two-parameter model. I use the three-parameter model here because it reveals the substantive shortcomings of analyses of complex allometry.

Next, I followed the protocol introduced by Sartori and Ball (2009) to examine the three-parameter power equation more closely. The treatment begins with the equation for describing the arithmetic distribution,
(3)

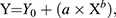
where the parameter *Y_0_* is the intercept and *a* and *b* are the other two parameters in the equation. The equation can be re-expressed in logarithmic form as
(4)

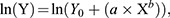
and the logarithmic representation can be rewritten as
(5)

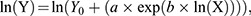
where exp(*b*×ln(X)) is the equivalent of X*^b^* in Eqn 4. By taking the first derivative for Eqn 5 (see http://www.derivative-calculator.net/), an expression is obtained for the conditional tangent, or slope, for the line described by the three-parameter power equation in logarithmic form, namely,
(6)

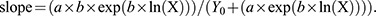
When *Y_0_* is −0.13, as in the case of the cockroaches (Table 2), the slope for the line fitted to logarithmic transformations varies as a function of ln(X) (Fig. 1C) and the power function itself follows a curvilinear path in the log–log plot (Fig. 1A). On the other hand, when *Y_0_* is zero, the right side of Eqn 6 can be simplified to *b*, which means that the slope is a constant (Fig. 1D) and that the equation fitted to transformations defines a straight line (Fig. 1A). Back-transforming the equation for the straight line yields a two-parameter power equation of simple allometry on the arithmetic scale. Of course, this means that the curvilinearity in logarithmic domain is caused by the requirement for a non-zero intercept to describe the allometric relationship in arithmetic domain. This finding has general importance and applies to all situations in which a quadratic polynomial is used to describe a bivariate distribution for logarithmic transformations (Sartori and Ball, 2009; Packard, 2017b).

## DISCUSSION

The intercept, *Y_0_*, in the three-parameter power equation describing the untransformed distribution for metabolic allometry of Madagascan cockroaches does not differ significantly from zero (*P*=0.14), and the fitted model does not capture substantially more information than does the two-parameter model fitted to the same data (ΔAIC=4.8). Applied statisticians would likely conclude that the two-parameter equation is just as good as the three-parameter function (Richards, 2005; Burnham et al., 2011; Richards et al., 2011), or that the intercept in the three-parameter model is so near to zero that it can be safely disregarded with little loss of information. On the other hand, the quadratic term in the polynomial fitted to logarithmic transformations is a significant source of variation (*P*=0.003), and the model itself is better than the straight line fitted to the distribution (ΔAIC=10.2). The same statisticians would probably conclude that the quadratic equation is the better fit to transformations and that it should be accepted over the straight line (Richards, 2005; Burnham et al., 2011; Richards et al., 2011). Thus, examination of the untransformed observations leads to a different set of conclusions than does an analysis that begins with transformations: whereas the analysis of untransformed data points to a two-parameter power equation as the better fit to the original data, the examination of logarithms implies that a three-parameter function is needed. This outcome is part of the problem stemming from the use of transformations when they are not needed (Packard, 2020; 2023). Even a seemingly negligible non-zero intercept in a power equation in arithmetic domain can cause the model fitted to transformations to be significantly curvilinear.

Of even greater importance, however, is the interpretation that is attached to the quadratic term in the quadratic equation describing logarithms (Fig. 1A). First, using logarithmic transformations to fit a two-parameter equation to the original data indirectly (the Huxlian method) requires that the distribution be linear, and any departure from linearity obviates modeling the transformations as an equation of simple allometry (Reeve, 1940; Kavanagh and Richards, 1942; Richards and Kavanagh, 1945). Second, curvilinearity in log domain reflects a change in the slope of the line at each level for ln(X), but this change in scaling is caused by failure to identify the correct form for the equation in arithmetic domain. The change in the slope at each level for ln(X) is caused by the need for an intercept in the power equation describing untransformed observations (Gould, 1966) – not by a continuously changing exponent in that equation.

Complex allometry is probably more common than is generally recognized (Strauss, 1993). For example, many examples of polyphasic, loglinear allometry (*sensu* Strauss, 1993) are actually curvilinear distributions that could have been described just as well, or even better, by quadratic equations on the logarithmic scale (Lemaître et al., 2014) and by three-parameter power equations on the linear scale (Packard, 2015b). Interpretations of new research, and formulations of new or revised theoretical models for evolution of form and function, need to be based on actual patterns of variation in the bivariate data of interest. Such patterns are more likely to be identified correctly in studies that focus on untransformed measurements (Finney, 1989; Menge et al., 2018; Packard, 2020; 2023).

In conclusion, allometric variation in metabolic rate of Madagascan cockroaches is well characterized by a simple, two-parameter power equation, and not by the three-parameter power equation that is indicated by an examination of logarithmic transformations. The allometric exponent for the two-parameter equation is 0.52, which represents a substantial departure from the values of 0.67 and 0.75 that are of wide concern to students of allometry. However, the power function is merely an empirical equation that describes pattern and trend in the arithmetic distribution (Pantin, 1932) and should not be accorded importance greater than this. The general finding reported here is not unique to Madagascan cockroaches and applies, instead, to almost all reported cases complex allometry.

## Supplementary Material

10.1242/biolopen.060148_sup1 Supplementary information

Table S1.
