# Supplementary material for: What is complex allometry?
Source: Biol Open. 2023 Dec 21;12(12):bio060148. doi: 10.1242/bio.060148 (PMC10751937; doi:10.1242/bio.060148)
Supplement: Supplementary information [file biolopen-12-060148-s1.pdf]

Table S1.

Available for download at
https://journals.biologists.com/bio/article-lookup/doi/10.1242/bio.060148#supplementary-data

Biology Open • Supplementary information
